# Impact of switching to tenofovir alafenamide on weight gain as compared to maintaining a non-tenofovir alafenamide containing regimen

**DOI:** 10.1097/MD.0000000000027047

**Published:** 2021-08-27

**Authors:** Julia Darnell, Sonia Jain, Xiaoying Sun, Huifang Qin, Timothy Reynolds, Maile Young Karris, Lucas A. Hill

**Affiliations:** a Skaggs School of Pharmacy and Pharmaceutical Sciences, UC San Diego, San Diego, CA; b Herbert Wertheim School of Public Health, Biostatistics Research Center, UC San Diego, San Diego, CA; c Department of Medicine, UC San Diego, San Diego, CA; d Department of Medicine, Division of Infectious Diseases and Global Public Health, UC San Diego, San Diego, CA.

**Keywords:** BMI, HIV, metabolic complications, tenofovir alafenamide, weight

## Abstract

Evaluate the impact of switching to an anti-retroviral regimen containing tenofovir alafenamide (TAF) on weight and the development of metabolic complications compared to remaining on a non-TAF containing regimen.

Single-center retrospective case-control study.

We evaluated people living with human immunodeficiency virus (PLWH) who were on an anti-retroviral regimen not containing TAF and were switched to a regimen containing TAF between January 1, 2016 and September 30, 2018. The control group included PLWH on a TAF free regimen throughout the study period. The primary outcome was change in weight from baseline to 12 months postswitch. Secondary outcomes included percent change in weight, change in body mass index (BMI), change in BMI class, and new diagnoses of diabetes, hypertension, and hyperlipidemia (HLD) during the study period.

PLWH switched to TAF (n = 446) demonstrated significantly greater mean increase in weight compared to the control group (n = 162) (1.97 vs 0.88 kg, *P* = .01), however the effect was only seen in those switched from tenofovir disoproxil fumarate. Those that switched to TAF also had a significantly higher percent increase in weight, increase in BMI, and BMI class. We observed a higher rate of new diagnosis of HLD in the control group compared to the TAF switch group during the study period.

PLWH switched to TAF had greater increases in weight after 1 year as compared to those continuing on a TAF free regimen. However, this did not translate to higher rates of obesity related illnesses such as diabetes, hypertension, and HLD during the follow up period.

## 1 Introduction

Successful treatment of human immunodeficiency virus (HIV) with anti-retroviral therapy (ART) relies on selection of a regimen that is efficacious, well tolerated, and individualized to ensure minimal impact on HIV associated non-AIDS comorbidities and quality of life.^[1]^ Modern ART provides the option of single tablet regimens with high rates of viral suppression and limited side effects for the majority of people living with HIV (PLWH).^[2–4]^ The success of ART relies on adherence, a complex health behavior impacted by factors that include pill burden, accessibility and cost, health literacy, psychosocial barriers, and medication side effects.^[5]^ To address these barriers, HIV providers and pharmacists routinely monitor, provide education and manage medication side effects in PLWH. Ongoing evaluation and reporting of postmarketing anti-retroviral (ARV) side effects remain critical steps towards optimizing adherence.

The Department of Health and Human Services HIV Treatment Guidelines recommend 5 options for initial ART, 3 of which include either tenofovir alafenamide (TAF) or tenofovir disoproxil fumarate (TDF) in combination with emtricitabine (FTC) and an integrase strand transfer inhibitor (INSTI).^[1]^ The benefits of TAF compared to TDF include absence of a decline in bone mineral density and improved renal safety.^[6–8]^ The more favorable adverse effect profile of TAF is thought to be due to its pharmacokinetic features that lead to lower serum concentrations and higher intracellular concentrations of tenofovir compared to TDF resulting in a lower prevalence of off-target effects.^[9]^

Recent findings report weight gain associated with ART, primarily attributed to the INSTI class, with the highest increases in weight seen with the use of second generation integrase inhibitors dolutegravir (DTG) and bictegravir (BIC).^[10–12]^ However, studies in treatment naïve PLWH suggest TAF is also implicated in weight gain, with risk factors for greater weight gain being female sex and black race.^[11,12]^ The ADVANCE study, a South African based randomized controlled trial evaluating potential first line ARV regimens (DTG + TAF/FTC versus DTG + TDF/FTC versus efavirenz [EFV] + TDF/FTC), demonstrated that PLWH in the TAF group were at higher risk for developing obesity compared to the TDF groups.^[11]^ Despite a growing body of evidence demonstrating weight gain in PLWH starting INSTIs and TAF, the downstream clinical consequences and mechanisms remain unknown. However, it has been well established that obesity increases the risk for various diseases such as heart disease, hypertension (HTN), diabetes, kidney disease, and cancers.^[13–16]^ It is also unclear if switching virologically suppressed PLWH from a non-TAF to TAF containing regimen results in weight gain, with the evidence being limited to small studies that lack evaluation of metabolic complications related to possible weight gain and/or lack a comparator group.^[17,18]^ We performed a single center retrospective study to characterize the impact on weight and body mass index (BMI) in PLWH switched from a non-TAF to TAF based regimen compared to time-matched PLWH maintained on a non-TAF containing regimen over a 12 month period. We also compared the development of diabetes mellitus (DM), hyperlipidemia (HLD), and HTN during the study period between the 2 groups.

## 2 Methods

### 2.1 Study population

This was a single center, retrospective, observational case-control study conducted at the University of California, San Diego. Data were obtained from both the Center for AIDS Research Network of Integrated Clinical Systems and the local medical record. The UCSD Human Subjects Research Protection Program approved this study.

Included in the case group were PLWH who were on an ARV regimen that did not contain TAF and were switched to an ARV regimen containing TAF between January 1, 2016 and September 30, 2018. The control group included PLWH on a TAF free ARV regimen throughout the same study period. Based on variability in HIV provider documentation, we could not consistently identify reasons for ARV switching or not. Exclusion criteria was less than 1 year of follow up after change to a TAF based regimen, absence of weight and BMI data at both 6 and 12 months after change to a TAF based regimen, or a further switch in ART during the study period. Baseline characteristics collected included age, sex, ethnicity, baseline weight, baseline BMI, veterans aging cohort study (VACS) score, baseline CD4 count, baseline HIV viral load, current physical activity, active substance use, and history of diagnosis of metabolic co-morbidities (DM, HLD, HTN). Baseline was defined as the date of ARV switch for cases, and as the date of the first available data within the study period for controls. For physical activity, answers to 2 patient reported outcome questions were assessed: “Do you regularly engage in strenuous exercise or hard physical labor?” and “Do you exercise or labor at least three time a week?” If persons answered yes to either or both questions they were coded as physically active, if they responded no to both they were coded as not physically active. Regarding active substance use, data were gathered by patient reported outcomes for each substance: “In the last three months did you use: cocaine, amphetamines, street opiates, prescription opiates, sedative or sleeping pills, inhalants, prescription stimulants for non-medical use.” Answering yes to 1 or more of these questions was coded as yes for active substance use.

### 2.2 Outcomes measures

The primary outcome was change in weight 12 months after switching to a TAF based regimen as compared to change in weight 12 months from the start of the study period in the control group. Weight and BMI for the cases were assessed at 6 months prior to ARV switch, baseline, and 6 months and 1 year after initiation of TAF. Weight was collected during usual HIV clinic visits in the outpatient setting. The 6 month prior weight data were collected to assess if weight gain was a trend that started prior to the switch to TAF, versus after the switch. Weight and BMI for the control group were assessed every 6 months throughout the study period. We also evaluated the development of new DM, HLD, and HTN during the study period for those without a history of the diagnosis prior to or within 3 months after the start of the study period. All diagnosis data were obtained using diagnosis codes and date of diagnosis from the medical record.

### 2.3 Statistical analysis

We used descriptive analyses to compare baseline characteristics between the cases and controls. Fisher exact test was used to compare categorical variables, while Wilcoxon Rank Sum test was used to compare continuous variables. The primary analysis applied a linear mixed effects model to assess the change in weight between the study groups. The dependent variable was change in weight from baseline at 6 and 12 months. Fixed effects included study group, timepoint, group-by-timepoint interaction, age, sex, VACS score, and BMI at baseline. Random effects included random intercept. Baseline substance use and physical activity were not included in the model due to a higher rate of missing data with these variables. A sensitivity model was performed to compare the weight change of case groups with different class changes (INSTI to non-INSTI, INSTI to INSTI, non-INSTI to INSTI, non-INSTI to non-INSTI) against the control group. Additional sensitivity models were also conducted comparing the presence or absence of TDF prior to switching against the control group and comparing the initiation of different INSTIs in those not previously on an INSTI. The number of new diagnoses of DM, HLD, and HTN during the study period was compared between the 2 groups using Fisher exact test. Statistical analysis was conducted using statistical software R (version 3.6.1; The R Foundation, Vienna, Austria).

## 3 Results

### 3.1 Baseline demographics

**Figure 1:**
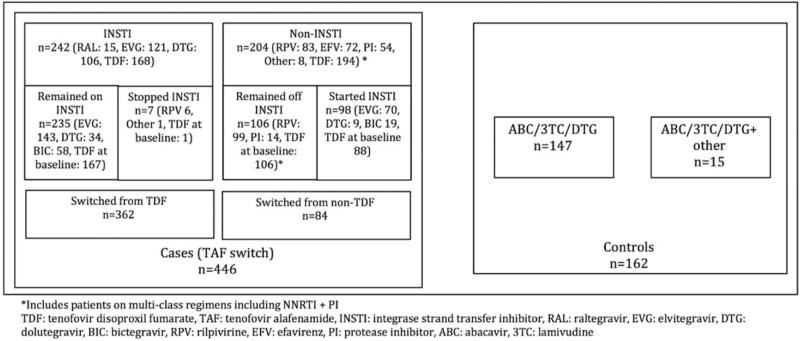
Detailed summary of groups evaluated for change in weight 12 months after start of study period.

A total of 608 PLWH were included in this study, of which 446 switched to a TAF containing regimen, and 162 continued on a non-TAF containing regimen during the study period. At baseline, the TAF group was younger, had a lower VACS score, and were more likely to report recent substance use. In regard to baseline comorbidities, a higher rate of HTN and HLD occurred in the control group (Table 1). All PLWH in the control group used a regimen that contained abacavir/lamivudine/DTG, and 15 (9.3%) were prescribed an additional ARV (7 rilpivirine [RPV], 6 darunavir/cobicistat, 1 atazanavir/cobicistat, 1 maraviroc). In the group that switched to TAF, 362 (81.2%) were switched from a regimen containing TDF, and 242 (54.3%) were on an INSTI containing regimen at baseline (Fig. 1).

**Table 1:** Comparison of baseline demographics.

Characteristic	TAF switch group (n = 446)	Control group (n = 162)	*P* value
Mean age (95%CI)	49.4 (48.4–50.4)	54.1 (52.5–55.7)	**<.001**
Female sex (%)	56 (12.6)	20 (12.4)	>.999
Transgender (%)	2 (0.45)	2 (1.23)	.29
Race/Ethnicity (%)			
White	189 (42.4)	74 (45.7)	.12
Black	51 (11.4)	29 (17.9)	
LatinX	161 (36.1)	47 (29.0)	
Asian	23 (5.2)	8 (4.9)	
Other/Unknown	22 (4.9)	4 (2.5)	
Undetectable HIV viral load (%)	409 (92.1)	147 (90.7)	.62
Mean CD4 count (cells/mm^3^) (95%CI)	655 (637–693)	644 (597–692)	.79
Mean VACS score (95%CI)	17.7 (16.3–19.1)	27.3 (24.1–30.5)	**<.001**
Substance use (%)			**.02**
Yes	166 (37.2)	47 (29.0)	
No	110 (24.7)	54 (33.3)	
Unknown	170 (38.1)	61 (37.7)	
Physical activity (%)			.28
Yes	134 (30.0)	35 (21.6)	
No	281 (63.0)	96 (59.3)	
Unknown	31 (7.0)	31 (19.1)	
Mean baseline weight (kg) (95%CI)	81.2 (79.8–83.0)	81.6 (78.9–84.4)	.84
Mean baseline BMI (95%CI)	26.8 (26.3–27.3)	27.1 (26.3–27.9)	.56
BMI category (%)			.79
Underweight	6 (1.4)	1 (0.62)	
Normal	170 (38.1)	58 (35.8)	
Overweight	187 (41.9)	68 (42.0)	
Obese	83 (18.6)	35 (21.6)	
Baseline diabetes (%)	59 (13.2)	31 (19.1)	.07
Baseline hypertension (%)	160 (35.9)	78 (48.1)	**.007**
Baseline hyperlipidemia (%)	35 (7.9)	81 (50.0)	**<.001**

BMI = body mass index, HIV = human immunodeficiency virus, TAF = tenofovir alafenamide, VACS = Veterans Aging Cohort study.

### 3.2 Switching to a TAF containing regimen is associated with weight gain

Persons that switched to a TAF containing regimen demonstrated significantly higher mean weight gain compared to controls at 6 (1.55 vs 0.19 kg, *P* < .001) and 12 months (2.02 vs 0.77 kg, *P* < .001) postswitch. This trajectory differed from the mean weight change (95%CI) of 0.13 kg (–0.23 to 0.49 kg) in the 6 month period prior to switching to a TAF containing regimen. Adjusting for age, sex, VACS score, and BMI at baseline demonstrated that mean change in weight at 6 (1.51 vs 0.30 kg, *P* = .005) and 12 (1.97 vs 0.88 kg, *P* = .011) months remained significantly higher for PLWH switched to TAF compared to controls. The adjusted percent change in weight and mean increase in BMI were also significantly higher for those switched to TAF (Table 2). When evaluating those that had an increase, no change, or decrease in BMI class (underweight, normal, overweight, obese) at 12 months, we observed a significantly higher proportion of PLWH with an increase in BMI class in those that switched to TAF as compared to controls (increase 18.2% vs 9.9%, no change 76.7% vs 82.7%, decrease 5.2% vs 7.4%, *P* = .03).

**Table 2:** Comparison of weight and BMI using linear mixed effects model adjusted for age, sex, VACS score, and BMI at baseline.

	TAF switch group (n = 446)	Control group (n = 162)	*P* value
Mean change in weight (kg) (95%CI)			
6 months	1.51 (1.08–1.93)	0.30 (−0.41–1.02)	**.005**
12 months	1.97 (1.55–2.40)	0.88 (0.17–1.60)	**.011**
Percent change in weight (95%CI)			
6 months	1.98 (1.47–2.48)	0.48 (−0.38–1.33)	**.003**
12 months	2.68 (2.18–3.19)	1.34 (0.49–2.20)	**.009**
Mean change in BMI (95%CI)			
6 months	0.51 (0.36–0.66)	−0.01 (−0.27–0.24)	**<.001**
12 months	0.70 (0.55–0.85)	0.15 (−0.11–0.40)	**<.001**

BMI = body mass index, TAF = tenofovir alafenamide, VACS = Veterans Aging Cohort study.

We did not observe significant differences in mean weight gain between females and males 12 months after switching to TAF (2.47 vs 1.95 kg, *P* = .45). We also observed no significant difference in weight gain based on race and ethnicity 12 months after switching to TAF. In the linear mixed effects model, factors that did impact risk for greater weight gain other than switching to TAF included younger age (*P* = .042) and lower baseline BMI (*P* < .001).

### 3.3 Linear mixed effects models demonstrated weight gain patterns differed based on concurrent ARVs in persons switching to TAF

Within the TAF switch group only persons that changed from a non-INSTI to INSTI (n = 98) and those that remained on a non-INSTI (non-INSTI to non-INSTI) regimen (n = 106) demonstrated greater mean 12 month weight gain compared to controls. Of those in the non-INSTI to non INSTI group, 71 (67%) were on a RPV containing regimen and all continued on RPV, 24 (22.6%) were on an EFV containing regimen and all switched to RPV, and 11 (10.4%) were on a boosted protease inhibitor containing regimen of which 3 switched to RPV and 8 remained on a boosted protease inhibitor. The greatest amount of weight gain at 12 months was observed in those in the non-INSTI to non-INSTI group. In the non-INSTI to INSTI group, the greatest amount of weight gain at 12 months was seen in those that initiated DTG (n = 9) as compared to elvitegravir (n = 70) and BIC (n = 19) (Table 3). There was no statistically significant difference in weight gain at 12 months when directly comparing those that initiated DTG vs elvitegravir (*P* = .07) and DTG vs BIC (*P* = .20) along with TAF. Additionally, a significant difference in mean weight gain at 12 months as compared to controls occurred in those that switched from a TDF based regimen to a TAF based regimen, but not in those that were not on TDF at baseline before switching to TAF (Table 3). Interestingly, the 2 groups that demonstrated a significant increase in mean weight compared to controls (non-INSTI to non-INSTI, and non-INSTI to INSTI) had significantly higher proportions of PLWH on baseline TDF when compared to the groups with non-significant weight gain (INSTI to INSTI, and INSTI to non-INSTI) (95.1% vs 69.4%, *P* < .00001).

**Table 3:** Sensitivity analysis comparing mean change in weight (kg) at 12 months using linear mixed effects model^∗^ adjusted for age, sex, VACS score, and BMI at baseline.

	TAF switch group	Control group (n = 162)	*P* value
INSTI to INSTI (n = 235) vs control (95%CI)	1.45 (0.88–2.03)	0.87 (0.16–1.58)	.21
INSTI to non-INSTI (n = 7) vs control (95%CI)	0.32 (−3.08–3.72)	0.87 (0.16–1.58)	.76
Non-INSTI to INSTI (n = 98) vs control (95%CI)	2.38 (1.48–3.27)	0.87 (0.16–1.58)	**.01**
Non-INSTI to non-INSTI (n = 106) vs control (95%CI)	2.89 (2.02–3.75)	0.87 (0.16–1.58)	**<.001**
Baseline TDF (n = 362) vs control (95%CI)	2.39 (1.92–2.86)	0.85 (0.14–1.56)	**<.001**
No baseline TDF (n = 84) vs control (95%CI)	0.25 (−0.73–1.23)	0.85 (0.14–1.56)	.32
Non-INSTI to EVG + TAF (n = 70) vs control (95%CI)	1.87 (0.80–2.93)	0.83 (0.14–1.53)	.12
Non-INSTI to DTG + TAF (n = 9) vs control (95%CI)	4.74 (1.81–7.68)	0.83 (0.14–1.53)	**.01**
Non-INSTI to BIC + TAF (n = 19) vs control (95%CI)	2.39 (0.36–4.43)	0.83 (0.14–1.53)	.16

BIC = bictegravir, BMI = body mass index, DTG = dolutegravir, EVG = elvitegravir, INSTI = integrase strand transfer inhibitor, TAF = tenofovir alafenamide, TDF = tenofovir disoproxil fumarate, VACS = Veterans Aging Cohort study.

∗ Conducted using 3 separate models (impact of class change, switching from TDF-based regimen, impact of initiation of different INSTIs).

### 3.4 TAF associated weight gain was not associated with obesity related disease

There was no significant difference in new diagnoses of diabetes (2.33% vs 5.34%, *P* = .14) and HTN (12.24% vs 20.24%, *P* = .07) between the TAF switch group and the controls during the study period. However, those switched to TAF had significantly lower incidence of HLD during the study period as compared to the control group (11.44% vs 35.8%, *P* < .001).

## 4 Discussion

Among our cohort of PLWH who were switched to a TAF containing ARV regimen, there was a significant increase in the amount of weight gained, percent change in weight, and increase in BMI as compared to the control group at 6 and 12 months. The mean increase in weight, as well as percent change in weight in our cohort is similar to those seen in previously reported cohorts after switching to a TAF containing regimen.^[17,18]^ In our cohort, we did not observe a signal of increasing weight 6 months prior to the switch, but did observe a mean increase of 1.55 kg 6 months after the switch suggesting an acceleration in weight gain after switching to TAF. Interestingly, all of our controls remained on a regimen containing DTG, which is known to contribute to weight gain, yet still demonstrated a significantly lower weight gain than PLWH who switched to TAF.^[10–12]^ We also did not observe significant differences in weight gain based on sex or race/ethnicity as observed in trials of treatment naïve individuals starting TAF suggesting patterns of weight gain may differ in scenarios of switching ARV regimens.^[11,12]^

After controlling for differences in age, sex, baseline VACS score, and baseline BMI we still observed significantly greater weight gain in those switched to TAF compared to the control group. Therefore, despite the control group being older with a higher VACS score, and therefore likely living with more comorbidities, these differences did not appear to drive the differences in weight gain between the 2 groups. We attempted to also include data related to physical activity and substance use at baseline. There was no statistically significant difference between the 2 groups at baseline in regards to physical activity suggesting this did not impact the differences in weight gain. However, we cannot rule out changes in physical activity during the duration of the study that may have impacted findings. The group that switched to TAF reported a higher frequency of substance use at baseline, however we did not have more specific data regarding specific substances and frequency of use, nor did we have data documenting discontinued or new incidence of substance use throughout the study. Thus, we could not draw conclusions as to the impact of substance use on changes in weight in the context of ARV switching.^[19]^

We also attempted to evaluate whether switching more than 1 ARV had effects on weight gain. Perhaps to be expected, given the known impact of INSTI on weight gain, those that were not on an INSTI and were switched to TAF and an INSTI at the same time did see a significant increase in weight as compared to controls at 12 months.^[10–12]^ Given that all controls were on DTG throughout the study period, we cannot formally test the interaction effect of specific INSTI and TAF on weight gain. However, the cases who switched from a non-INSTI containing regimen to TAF + DTG did experience the largest amount of weight gain as compared to those switched from non-INSTI to TAF + elvitegravir or TAF + BIC, although the difference did not reach statistical significance given the small sample sizes. To some extent this agrees with previous data demonstrating second generation INSTI are associated with the greatest amount of weight gain among the INSTI class, although the ability to draw conclusions is limited by small sample sizes of those not on INSTI pre-switch who were started on DTG and BIC in addition to TAF.^[10–12]^ A somewhat unexpected finding was that the greatest amount of weight change at 12 months occurred in those that remained on an INSTI free regimen and were changed to TAF. This suggests that changes in weight in the TAF switch group were not driven solely by the INSTI class. The majority of PLWH in this group were on a RPV containing regimen and all continued on RPV after switching to TAF, or were on an EFV containing regimen and switched to RPV along with TAF. Among the non-nucleoside reverse transcriptase inhibitors, RPV demonstrates the potential to contribute to more weight gain, whereas EFV may be more weight stabilizing.^[12,20]^ We also observed that a significant difference in weight gain only occurred in those on TDF at baseline who were switched to TAF. In the groups mentioned above, those that saw a significant increase in mean weight compared to controls also contained a significantly higher proportion on TDF at baseline. This suggests the changes in weight after switching to TAF were primarily due to removal of the weight stabilizing/weight loss effect of TDF containing regimens.^[21]^ Unfortunately, the mechanism of action for weight gain associated with specific ARVs and the weight stabilizing effect of other ARVs remains undescribed. Thus, we can only speculate on why weight gain after switching to TAF was only seen in the non-INSTI to non-INSTI and non-INSTI to INSTI groups. Our findings add to a growing body of work that support ongoing mechanistic studies of ART associated weight gain.

The finding of a significantly higher percentage of PLWH in the control group with baseline HTN and HLD was unexpected, but may be explained by a combination of factors. First, persons in the control group were older, with HTN and HLD being age associated chronic conditions.^[22]^ Second, those in the control group were on non-TDF containing regimens of abacavir/lamivudine/DTG with or without other ARVs, therefore this may have pre-selected PLWH at risk for non-AIDS co-morbidities, such as chronic kidney disease and osteoporosis. This is supported by a higher baseline VACS score in the controls. In addition, TDF is associated with decreases in cholesterol, and this effect may have contributed to differences in the presence of baseline HLD between the 2 groups.^[23]^ Interestingly, we observed a higher rate of new diagnosis of HLD during the study period in the control group as compared to the TAF switch group. Again, this may be a result of the variables described above.

We did observe clear weight gains in PLWH who switched to TAF compared to those who did not, and do view these changes as clinically meaningful because we concurrently observed in the same group a significantly higher proportion of persons with an increase in their BMI class. This study cannot determine if the weight gain associated with switching to TAF outweighs the benefit of TAF over TDF in terms of reduction in risk to bone and kidney health.^[6–8]^ However, this study along with the growing body of work associating weight gain with TAF should prompt careful consideration and counseling when determining the optimal ARV regimen for PLWH, particularly as it relates to optimizing adherence by minimizing AEs based on individual preferences.^[5]^

Limitations of our study include a retrospective study design limiting the ability to control for differences between the 2 cohorts. However, we attempted to control for this statistically by comparing weights between the 2 groups using a linear mixed effects model adjusting for several baseline variables. That being said, we did not document the duration our control subjects were on their current ARV regimen prior to the study period which may impact their weight gain trajectory.^[10]^ Additionally, we only have weight data up to 12 months after the switch to TAF, and the trajectory of weight gain when switched from a non-TAF to TAF based regimen beyond 12 months is still unknown. While we did not observe differences in weight gain due to gender or race, our cohort is predominately white males. We also were only able to report the viral suppression rates and CD4 counts at baseline, however the 2 cohorts were similar, both with greater than 90% of subjects with viral suppression and high mean CD4 counts demonstrating this is a population of subjects with well controlled HIV. Finally, the presence of baseline metabolic comorbidities was not evenly distributed between the cohorts, suggesting differences in baseline factors that may lead to an unequal development of new diagnoses during the study period. It is unknown how additional follow up time would impact the development of further metabolic complications.

In conclusion, we observed a significant difference in the increase in weight after 12 months in PLWH switched from a non-TAF to a TAF containing regimen as compared to PLWH maintained on a TAF free regimen. Weight change was greatest in those switched to TAF who continued on a non-INSTI containing regimen and appears to be primarily driven by the switch from TDF to TAF. We did not observe a greater proportion of new diagnosis of diabetes, HTN, or HLD in those switched to TAF during the study period.

## Author contributions

**Conceptualization:** Julia Darnell, Maile Young Karris, Lucas Hill.

**Data curation:** Julia Darnell, Huifang Qin, Timothy Reynolds, Lucas Hill.

**Formal analysis:** Julia Darnell, Sonia Jain, Xiaoying Sun, Maile Young Karris, Lucas Hill.

**Methodology:** Julia Darnell, Sonia Jain, Xiaoying Sun, Maile Young Karris, Lucas Hill.

**Project administration:** Lucas Hill.

**Supervision:** Maile Young Karris, Lucas Hill.

**Validation:** Maile Young Karris, Lucas Hill.

**Writing – original draft:** Julia Darnell, Sonia Jain, Xiaoying Sun, Huifang Qin, Timothy Reynolds, Maile Young Karris, Lucas Hill.

**Writing – review & editing:** Julia Darnell, Sonia Jain, Xiaoying Sun, Huifang Qin, Timothy Reynolds, Maile Young Karris, Lucas Hill.
